# A Systematic Review of Imaging Studies in the Combined and Inattentive Subtypes of Attention Deficit Hyperactivity Disorder

**DOI:** 10.3389/fnint.2020.00031

**Published:** 2020-06-24

**Authors:** Jacqueline Fifi Saad, Kristi R. Griffiths, Mayuresh S. Korgaonkar

**Affiliations:** ^1^Brain Dynamics Centre, Westmead Institute for Medical Research, Westmead Hospital, Sydney, NSW, Australia; ^2^The Discipline of Psychiatry, Sydney Medical School, The University of Sydney, Camperdown, NSW, Australia

**Keywords:** attention deficit hyperactivity disorder (ADHD), clinical subtypes, predominantly inattentive type, combined type, brain networks, neuroimaging

## Abstract

**Objective:** Insights to underlying neural mechanisms in attention deficit hyperactivity disorder (ADHD) have emerged from neuroimaging research; however, the neural mechanisms that distinguish ADHD subtypes remain inconclusive.

**Method:** We reviewed 19 studies integrating magnetic resonance imaging [MRI; structural (sMRI), diffusion, functional MRI (fMRI)] findings into a framework exploring pathophysiological mechanisms underlying the combined (ADHD-C) and predominantly inattentive (ADHD-I) ADHD subtypes.

**Results:** Despite equivocal structural MRI results, findings from fMRI and DTI imaging modalities consistently implicate disrupted connectivity in regions and tracts involving frontal striatal thalamic in ADHD-C and frontoparietal neural networks in ADHD-I. Alterations of the default mode, cerebellum, and motor networks in ADHD-C and cingulo-frontoparietal attention and visual networks in ADHD-I highlight network organization differences between subtypes.

**Conclusion:** Growing evidence from neuroimaging studies highlight neurobiological differences between ADHD clinical subtypes, particularly from a network perspective. Understanding brain network organization and connectivity may help us to better conceptualize the ADHD types and their symptom variability.

## Introduction

Neurobiological research in attention deficit hyperactivity disorder (ADHD) has witnessed exponential growth over the past two decades, uncovering key brain features underlying functional deficits in response inhibition, hyperactivity, and inattention typically observed in individuals with this disorder. These advances are important as ADHD has an estimated global prevalence of 3.4% in children and adolescents (Polanczyk et al., 2015). The Diagnostic and Statistical Manual of Mental Disorders (DSM-V) classifies three presentation types (i.e., “subtypes”) of ADHD: predominantly inattentive type (ADHD-I), predominantly hyperactive/impulsive type (ADHD-HI), or combined type (ADHD-C) (DSM-V, 2013).

The evolution of ADHD subtypes over updated editions of the DSM highlights the historical challenge encompassing categorical subgrouping due to the heterogeneity of ADHD symptoms among individuals. ADHD subtyping was first introduced in DSM-III to separate individuals with hyperactivity from those without hyperactivity, following which it was then abandoned in DSM-III-R, and later reintroduced in DSM-IV as ADHD-I, ADHD-C and with an additional third subtype, ADHD-HI (Barkley, 2014). The current DSM-V (DSM-V, 2013) edition retained the three-subtype model, now termed “presentations,” to better account for symptom variation (Faraone et al., 2015). However, these inhomogeneous DSM subtype categories continually spar debate among researchers adjunct to the limited knowledge of the neural mechanisms, which underlie the ADHD subtypes (Lange et al., 2014). For instance, both the ADHD-C and ADHD-I types experience academic and social difficulties, but with variations in the presentation of these clinical symptoms. For example, impairment in social functioning and response inhibition, in addition to comorbid externalizing features, i.e., oppositional defiant and conduct disorders, tends to be associated with the ADHD-C type. Meanwhile, significantly higher levels of shyness and passive social behavior is more often observed in those with the ADHD-I type, together with a greater prevalence of internalizing comorbid disorders such as anxiety, depression, and self-esteem difficulties (Baeyens et al., 2006; Willcutt et al., 2012). Furthermore, clinical symptoms of hyperactivity and impulsivity tend to no longer meet diagnostic criteria in late adolescent and adult ADHD despite the enduring nature of inattentive symptoms (Faraone et al., 2015). Thus, the ADHD-C type appears diagnostically more unstable over development than the ADHD-I type, which seems to suggest differing brain organization may underlie these two ADHD types. Consequently, this presents a diagnostic challenge, which at best measures symptoms that are present at that moment in time (Nigg et al., 2010).

To address these challenges involving the diagnostic conceptualization of ADHD types, magnetic resonance imaging (MRI)-based research has made attempts to establish clear neurobiological pathways of the ADHD subtypes utilizing measures of cortical functional activation, structural volumes, and more recently, by studying brain connectivity and networks. The proposal of a neurocircuitry-based model in ADHD incorporates knowledge on the role of inter-regional network organization involving frontal, temporal, and parietal regions, indicating the default mode network (DMN) and cingulo-frontal parietal (CFP) attention network to underlie the ADHD types (Bush, 2010; Castellanos and Proal, 2012; Fair et al., 2012; De La Fuente et al., 2013).

Emerging evidence suggests that connectivity differences may better conceptualize ADHD subtypes and explain its variations in functional symptoms, which have led to an increase in the adoption of network-based analyses in the ADHD literature (Cao et al., 2014). This paradigm shift posits that the clinical symptoms of ADHD may, in fact, result from dysfunctional network connections rather than discrete structural or functional abnormalities. Individual differences in connectivity profiles may also underlie the different clinical symptoms associated with each subtype of ADHD.

Several systematic reviews and meta-analyses of ADHD neuroimaging studies are available (Seidman et al., 2005; Paloyelis et al., 2007; Castellanos and Proal, 2012; Cortese et al., 2012; Kasparek et al., 2013; Matthews et al., 2014; Faraone et al., 2015) with recent reviews of network properties (Bush, 2010; Konrad and Eickhoff, 2010; De La Fuente et al., 2013; Cao et al., 2014; Posner et al., 2014) highlighting the current trend in brain connectome research. However, with the exception of a systematic review evaluating the validity of DSM-IV symptom domains and neuroimaging findings in ADHD and subtypes (Willcutt et al., 2012), a comprehensive systematic review of neuroimaging measures of ADHD subtypes remains unavailable in the literature. Thereby, the goal of this article was to systematically review the literature and consolidate data from a range of neuroimaging studies, limited to pediatric populations to evaluate whether the observed differences between the clinical subtypes of ADHD exist consistently across a diverse range of modalities. In the following sections, first, we summarize the findings from studies employing structural MRI (sMRI), diffusion tensor imaging (DTI), followed by resting state (rs-fMRI) and task-based functional MRI (fMRI). Also, within these sections, wherever possible, we describe network-based findings relative to the neuroimaging measure examined. Finally, we discuss the implications of these results for future research but also in the context of further clarifying ADHD subtype pathophysiology.

## Methods

This systematic review has been prepared in accordance with the recommended reporting items for systematic review and meta-analysis protocols (PRISMA) 2015 checklist to include in a systematic review protocol (Shamseer et al., 2015).

### Information Source and Search Strategy

**Figure 1:**
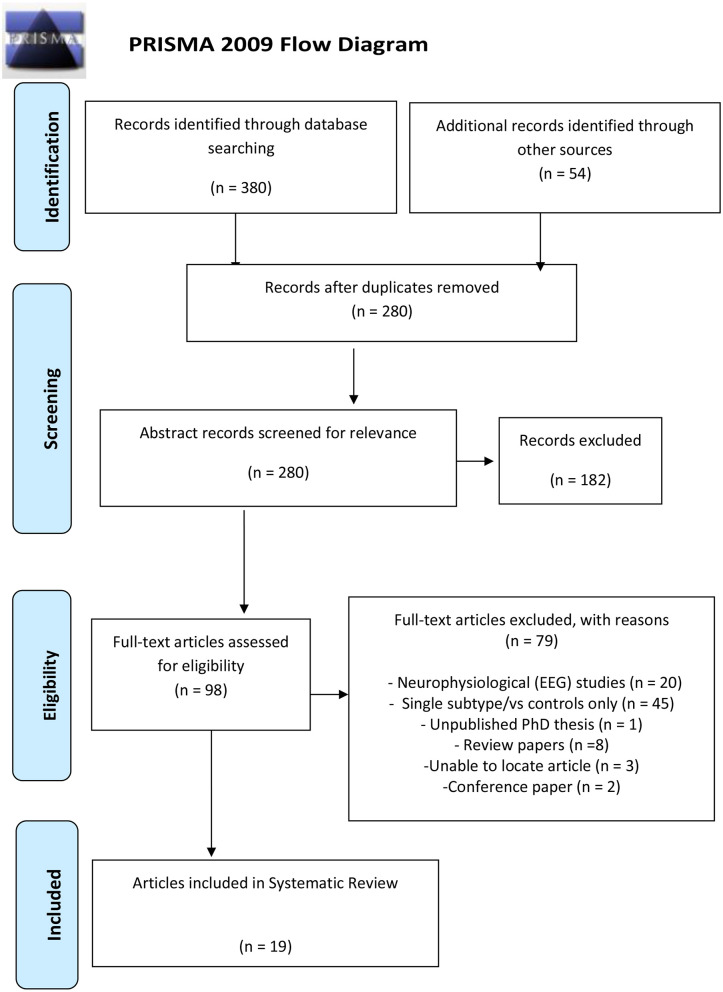
Prisma flowchart of the study selection process.

A systematic search of the databases was conducted utilizing PubMed, Google Scholar, Medline, Web of Science, and PsycINFO to identify relevant published literature to address the research objective. The Cochrane Library was searched to check for any previously registered reviews. The reference lists from identified articles including review papers were also searched for relevant articles. The literature search was performed by a single investigator JFS and cross-checked by MSK. The search terms/keywords used included the following: (ADHD or Attention deficit hyperactivity disorder) AND (subtype or combined presentation or inattentive presentation) AND (MRI or fMRI or functional connectivity or graph network or DTI or diffusion tensor imaging). The study selection process was conducted in accordance with PRISMA (preferred reporting items for systematic reviews and meta-analyses) criteria (Moher et al., 2009), illustrated in Figure 1. Each selected article was also entered into Endnote (reference management database) including the search term and engine that located each article. This search was limited to English-language articles published from 1/01/1995 to 19/06/2018.

### Study Selection Criteria

The study selection process is illustrated in Figure 1. Following the removal of duplicates, articles were screened based on the inclusion and exclusion criteria for papers that were of relevance to the research question. The review article aimed to include comparative studies of any design examining the structural and functional features from DSM-IV, DSM-IV-TR, or DSM-V defined ADHD-C type and ADHD-I subtypes. We considered the following modalities to address the research question: structural MRI, diffusion tensor imaging (DTI), resting state (rs-fMRI), and task-based functional MRI (task-fMRI). These neuroimaging modalities are briefly described below in each result section.

### Exclusion Criteria

Studies that were non-subtype/presentation specific, based on an adult ADHD sample (i.e., above 21 years old), focused on the evaluation of pharmacological treatment, written in a language other than English, or review papers were excluded (although we carefully screened available reviews for any eligible articles). Full-text articles were then screened based on the exclusion criteria. Articles that met the eligibility criteria and were included in this review article are summarized in Table 1.

**Table 1:** Summary table of neuroimaging studies on patients with ADHD combined and inattentive subtypes.

**References**	**ADHD-C (Female, %) Mean ± SD**	**ADHD-I (Female, %)Mean ± SD**	**Controls**	**Age Range (years)**	**Imaging modality measures**	**Analysis/measures**	**Key findings**
Al-Amin et al. (2018)	*N* = 196 37 (19%) 10.98 ± 2.92	*N* = 131 36 (27%) 11.95 ± 2.55	*N* = 553	7–21	Structural	Voxel-wise	Reduced hippocampal volume in ADHD-C relative to ADHD-I and controls
Vilgis et al. (2016)	*N* = 33 0 12.71 ± 2.06	*N* = 15 0 12.30 ± 2.56	*N* = 31	8–17	Structural	Voxel-wise	No significant differences in GM or WM between ADHD-I and ADHD-C
Pineda et al. (2002)	*N* = 15 8 (54%) 9.3 ± 1.3	*N* = 15 8 (54%) 9.3 ± 1.3	*N* = 15	6–11	Structural	Voxel-wise	No significant differences in the caudate nucleus head between both ADHD subtypes and controls
Semrud-Clikeman et al. (2014a)	*N* = 25 3 (12%) 14 ± 2.09	*N* = 22 3 (14%) 14.97 ± 2.23	*N* = 27	9–16	Structural	Shape analysis methodology using the FAST tool from FSL	Bilaterally smaller volumes of the caudate and ACC in ADHD-C relative to ADHD-I and controls
Carmona et al. (2005)^a^	*N* = 15 4 (27%) 10.7 ± 3.39	*N* = 5 0 11.63 ± 2.04	*N* = 25	6–16	Structural	Voxel-wise	No significant differences in GM or WM between ADHD-I and ADHD-C
Carmona et al. (2009)^b^	*N* = 25 5 (20 %) 10.84 ± 2.6	*N* = 11 1 (9%) 12.72 ± 2.5	*N* = 42	6−1 8	Structural	Voxel-wise	No significant differences between the ADHD subtypes for absolute or relative volumes
Saad et al. (2017)	*N* = 18 4 (25%) 12.81 ± 2.85	*N* = 16 5 (28%) 13.70 ± 2.67	*N* = 28	8–17	Structural	Voxel based Graph Theory Structural covariance	No significant differences in GM. ADHD-I greater nodal degree in regions associated with limbic, visual and ventral attention networks involving the left hippocampus and calcarine and the right superior occipital and supramarginal gyrus. ADHD-C higher degree distribution in the cerebellum
Anderson et al. (2014)	*N* = 159 37 (23%) -	*N* = 109 36 (33%) -	*N* = 472	7.1–21.8	Structural	Non-Negative Matrix Factorization models	Structural graph theory network measures of the default mode network differed in ADHD-I relative to ADHD-C and controls
Svatkova et al. (2016)	*N* = 13 3 (24%) 12.78 ± 2.33	*N* = 20 4 (25%) 14.96 ± 2.37	*N* = 27	9–16	DTI	TBSS (FA, RD, AD, MD)	ADHD- I increased RD in the forceps minor relative to ADHD-C
Ercan et al. (2016a)	*N* = 24 0 10.5 ± 1.7	*N* = 24 6 (25%) 11.1 ± 2.0	*N* = 24	8–15	DTI	TBSS (FA, RD, AD)	Increased RD bilaterally and increased AD in brain regions mostly on the left side linked to fronto-striato-cerebellar regions in ADHD- C than ADHD-I
Lei et al. (2014)	*N* = 28 *3 (11%)* 9.3 ± 1.3	*N* = 28 *3 (11%)* *9.3* ± 1.3	N = 28	7–13	DTI	Voxel based analysis(FA, RD, AD)	Significant differences in ADHD-C relative to ADHD-I involving the motor circuit, increased FA and RD in the right thalamus, increased AD in the left post-central gyrus and right caudate and increased RD in the left postcentral gyrus and supplementary motor area
Hong et al. (2014)	*N* = 39 6 (15.4%) 9.30 ± 2.47	*N* = 26 6 (23.1%) 9.78 ± 2.81	*N = 26*	6.3 – 15.9	DTI	Tractography, NBS (FA)	Decreased FA in the right lateralized network involving 17 brain regions in ADHD-C relative to ADHD-I, involving the superior frontal gyrus, anterior cingulate gyrus and supplementary motor areas
Solanto et al. (2009)	*N* = 11 4 (36%) 11.2 ± 1.9	*N* = 9 2 (22%) 10.7 ± 1.3	-	7–13	Task fMRI	Go/No-Go response inhibition task	Significant connectivity differences between ADHD subtypes were identified mainly in the frontal, cingulate, and parietal cortices and partially in the temporal, occipital cortices and cerebellum. Classifier accuracy for distinguishing between ADHD subtypes was 91.18 % for both gambling punishment and emotion task paradigms
Orinstein and Stevens (2014)	*N* = 23 *0* *14.7* ± 1.85	*N* = 18 *4 (22%)* *15.20*± 1.72	*N* = 20	12–18	Task fMRI	Three-stimulus auditory oddball attention task	No significant differences in task performance between the ADHD subtypes
Ahn et al. (2015)	*N* = 61 10 (16%)	*N* = 31 13 (42%)	*N* = 86	7–18	Rs-fMRI	Sparse reduced rank (SRR) spatial-temporal modeling framework in the frequency domain	Higher power spectra in the right middle and inferior frontal gyrus in ADHD-C and higher power spectra in the left middle frontal gyrus and cingulate gyrus, right insula and right postcentral gyrus in ADHD-I
dos Santos Siqueira et al. (2014)	*N* = 110 25 (23%) 12.08 ± 2.55	*N* = 159 29 (18%) 11.24 ± 3.05	*N* = 340	7–14	Rs-fMRI	Graph Theory	Atypical connectivity patterns in the sensorimotor and DMN in ADHD-C and frontoparietal network and cerebellar regions in children with ADHD-I
Fair et al. (2012)	*N* = 112 22 (19%) 10.31 -	*N* = 80 22 (27%) 11.45 -	*N* = 455	7–14	Rs-fMRI	Seed, Graph Theory	Alterations of the DMN and the insular cortex in ADHD-C and aberrant network properties for the dorsolateral prefrontal cortex and cerebellum in ADHD-I
Pikusa and Jonczyk (2015)	*N* = 109 28 (23%) 12.05 ± 1.77	*N* = 158 24 (18%) 11.24 ±3.25	*N* = 478	7–14	Rs-fMRI	Fractional amplitude low-frequency fluctuations	No significant differences in FALFF in Broca areas (Brodmann areas 44/45) between the two ADHD types
Sanefuji et al. (2016)	*N* = 68 *15 (22%)* *11.46* ± 2.8	*N* = 53 *19 (36 %)* *11.28* ± 2.7	*N* = 170	7–17	Rs-fMRI	Statistical clustering to define data-driven subtypes	Increased connectivity in the right ventral attention network in ADHD- I relative to ADHD-C and controls

*ADHD-C, Attention Deficit Hyperactivity Disorder-Combined type; ADHD-I, Attention Deficit Hyperactivity Disorder-Predominantly Inattentive type; AD, axial FAST, 3D brain image segmentation tool into different tissue types; FSL, analysis tools for FMRI, MRI and DTI brain imaging data. diffusivity as a variable of axonal maturation; DTI, Diffusion Tensor Imaging; FA, fractional anisotropy as a marker of microstructural architecture; FALFF, Fractional amplitude low-frequency fluctuations; GM, gray matter; MD, mean diffusivity; NBS, Network Based Statistics; RD, radial diffusivity to assess axonal myelination Rs-fMRI, resting state functional MRI; TBSS, Tract Based Spatial Statistics; WM, white matter.

### Data Extraction

Data extraction tools involved standardized database templates and forms using excel and Endnote X8. Quality check and accuracy of the first reviewer's (JFS) data extraction was performed by the second reviewer (MSK). Data extracted from the studies were study design, population source, the sample size, age range, location, gender, DSM edition, neuroimaging measures used, and study findings/outcome. Data were then organized into categories based on the type of neurobiological measure used.

### Summary Measures

#### Structural MRI

Magnetic resonance imaging (MRI) allows non-invasive measurements of neuroanatomical properties of the brain (Friedman and Rapoport, 2015). Statistical parametric mapping techniques, such as voxel-based morphometry (VBM) and surface-based modeling (e.g., using FreeSurfer) of T1-weighted anatomical scans, are commonly applied to quantify global and local measures of gray matter structures such as volume, cortical thickness, and surface area in the brain (Carmona et al., 2005).

#### Diffusion Tensor Imaging

Diffusion tensor imaging (DTI) is an MRI technique, which can provide information on the microstructural properties of brain white matter (WM) by observing the directionality and coherence of water diffusion (Matthews et al., 2014). DTI measures typically used are fractional anisotropy (FA) as a marker of microstructural architecture, radial diffusivity (RD) to assess axonal myelination, axial diffusivity (AD) as a variable of axonal maturation, and mean diffusivity (MD) to show the average degree of water diffusion in all directions (Alexander et al., 2007). Tract-based spatial statistics (TBSS) is an analysis using DTI-derived scalar 222 measures of fractional anisotropy (FA), mean (MD), radial (RD), and axial (AD) diffusivity as indices of water diffusion properties in WM tracts (Smith et al., 2006).

#### Functional MRI

Functional MRI allows non-invasive measurements of brain activation and mapping brain function by detection of alterations in blood oxygenation level (BOLD) changes in response to stimuli (task-based paradigms) or at resting state (rs-fMRI) (Posner et al., 2014). Resting state functional connectivity MRI (rs-fMRI) allows a measure of inter-functional connectivity between brain regions and understand brain functional networks (Cao et al., 2014).

#### Brain Network Topology

Global brain network topology may be derived using graph analysis measures of global and local efficiency, characteristic path lengths, and clustering coefficient to assess brain network integration. Nodal degree measures the number of connections a node has with the rest of the network. The nodes with a higher degree are considered to be highly interactive regions and densely distributed, which are indexed to their functional associations within and across brain networks (Sporns, 2013). Greater nodal degree may also reflect reduced efficiency of a network where greater connections than what is typically required are needed to relay information across the brain (Rubinov and Sporns, 2010). Network-based statistics (NBS) and graph theoretical analysis can be applied to assess structural connectivity, which is represented by anatomical connections formed by WM axonal fiber tracts to understand whether these connections underpin functional network connections (Sporns, 2013).

## Results

### Study Selection

The initial keyword search yielded 380 articles, 45 from PsycINFO, 81 from PubMed, 38 from Medline, 211 from Web of Science, and 5 from the Cochrane library. An additional search using Google Scholar yielded 54 articles. Of the 98 full-text articles assessed by the exclusion criteria, 79 articles were excluded as 20 articles were neurophysiological EEG studies, 45 articles included one subtype only, 8 were review articles, 1 was an unpublished Ph.D. thesis [investigating neurocognitive (neuropsychological) measures], 3 articles could not be located, and 2 conference papers (both investigating neurofeedback efficacy in ADHD subtypes). A total of 19 studies from the selection process were included in this study as illustrated in Figure 1.

### Study Characteristics

The characteristics and outcomes of the studies included in this review examining neural mechanisms in the clinical subtypes of ADHD are summarized in Table 1. Table 2 summarizes the main differences in structural and functional brain features between the ADHD combined and inattentive subtypes. Studies included a total of 4,851 (1,064 ADHD-C, 940 ADHD-I, and 2,847 controls) child and adolescent participants aged 6–21 years, which had both male and female (214 ADHD-C, 221 ADHD-I) participants; 2 were conducted in Australia, 14 in the United States, 1 in the Middle East, and 2 in Asia. Of these included studies, eight studies were based on structural MRI, four were DTI studies, two involved task-based fMRI (fMRI), and five studies utilized rs-fMRI. Participants were generally recruited from outpatient and pediatric clinics or community samples, with seven studies utilizing data obtained from the ADHD-200 database (Bellec et al., 2017). Based on the publication range specified for the included studies, 17 of the 19 studies were published in the last 10 years.

**Table 2:** Summary differences in structural and functional brain features between ADHD combined and inattentive subtypes.

	ADHD-C	ADHD-I
Structural MRI	Volumetric Non-Sig^*^ Default mode network, motor, frontoparietal	Volumetric Non-Sig^*^ Limbic, visual and ventral attention networks
fMRI	Frontostriatal thalamic and visual deficits in addition to aberrant DMN connectivity	Frontoparietal, cerebellar deficits and aberrant CFP attention network patterns
Dti	RD, AD, FA, fiber tracts differed involving the cerebellum, frontostriatal, and frontoparietal regions in ADHD-C relative to ADHD-I	

* *Non-Sig: non-significant*.

### Structural MRI and ADHD Subtypes

All the eight studies using sMRI to examine neuroanatomical differences in the ADHD-C and ADHD-I subtypes evaluated gray matter volumes (Pineda et al., 2002; Carmona et al., 2005, 2009; Anderson et al., 2014; Semrud-Clikeman et al., 2014a; Vilgis et al., 2016; Saad et al., 2017; Al-Amin et al., 2018), and one of these studies utilized graph theory measures of network topology properties computed from volumetric measures between the subtypes of ADHD (Saad et al., 2017). All eight studies classified participant subtype using the DSM-IV criteria. Five of the eight studies found no significant subtype differences in gray matter volumes (Pineda et al., 2002; Carmona et al., 2005, 2009; Vilgis et al., 2016; Saad et al., 2017). One study found smaller volumes of the caudate and ACC in ADHD-C relative to ADHD-I type (Semrud-Clikeman et al., 2014a). Another study by Anderson et al. (2014) found decreased volumes in regions associated with nodes of the default mode network (DMN) including the posterior cingulate, precuneus, and parahippocampal regions for the ADHD-I group relative to ADHD-C. Another study observed reduced hippocampal volume in the ADHD-C type relative to ADHD-I and controls (Al-Amin et al., 2018). Notably, most of the studies, except for the Anderson et al. (2014) and Al-Amin et al. (2018) studies, which utilized the ADHD-200 database, have relatively smaller sample sizes, which may account for the null findings due to limited statistical power. Moreover, methodological differences in the three studies reporting significant differences also must be considered, as the other studies applied VBM analyses, which require stringent corrections to account for type 1 error due to multiple voxel-wise comparisons involved (e.g., family-wise error). In contrast, the Semrud-Clikeman et al. (2014a) study applied shape analysis methodology using the FAST tool from FSL and analyzed mean volume measures of only the predefined basal ganglia structures between the subtypes. Further, participants from the Al-Amin et al. (2018) study were aged 7–21 years compared to the other seven studies' age range of up to 18 years.

To investigate brain network properties using anatomical measures, Saad et al. (2017) created a structural covariance network for each subtype group, defined as the Pearson correlation coefficient between gray matter volume measures of regions to perform graph theory analyses. The authors observed regional network differences, which showed ADHD-I participants, relative to ADHD-C, to have greater nodal degree distribution of the regions associated with the limbic, visual, and ventral attention networks involving the left hippocampus, the right superior occipital, and left calcarine and the right supramarginal gyrus, respectively. On the other hand, ADHD-C participants had a higher degree of distribution in the cerebellum, a region which is an important hub central to the motor network and is also known to interact with the frontoparietal executive control circuit.

### Diffusion Tensor Imaging and ADHD Subtypes

Of the four DTI studies available to review examining the microstructural properties of WM tracts between ADHD subtypes, three studies used whole brain voxel-wise analysis whereby two of these studies employed tract-based spatial statistical (TBSS) analyses of FA, RD, AD (Ercan et al., 2016a; Svatkova et al., 2016), and MD (Svatkova et al., 2016) diffusion properties, and one study analyzed FA, RD, and AD values using a voxel-based analysis (Lei et al., 2014). The fourth study utilized a whole brain connectome approach to map interregional brain connections using tractography and applied network-based statistic (NBS) analysis of FA values of these connections and also examined correlations of tract-averaged FA values and neuropsychological attention measures as a subsequent analysis (Hong et al., 2014).

The two studies, which employed tract-based spatial statistical (TBSS) analyses to examine differences of FA, RD, AD, and MD between the ADHD-C and ADHD-I subtype showed interesting findings. Svatkova et al. (2016) compared WM microstructure in 13 ADHD-C and 20 ADHD-I type children and found a single differential finding where ADHD-I had increased RD in the forceps minor relative to ADHD-C, which the authors suggest, corresponds to processing speed, a deficit more prominent in the inattentive type. Additionally, a number of differences were observed specific to subtype when compared to controls, with increased FA in the right cingulum in ADHD-C, and increased FA in ADHD-I involving regions linked to fronto–striatal–thalamic circuits and the cingulum bundle, which forms connections between the frontal, parietal and temporal lobes (Svatkova et al., 2016). The second study, by Ercan et al. (2016a) compared 24 ADHD-C and 24 ADHD-I children and adolescents with 24 controls and found increased RD bilaterally and increased AD in brain regions mostly on the left side linked to fronto–striato–cerebellar regions in ADHD-C than ADHD-I. The third study that used voxel-based analyses measured FA, RD, and AD values in children, aged 7–13 years old, and found significant differences in ADHD-C (*n* = 28), relative to ADHD-I (*n* = 28), involving the motor circuit, with increased FA and RD in the right thalamus, increased AD in the left postcentral gyrus and right caudate, and increased RD in the left postcentral gyrus and supplementary motor area (Lei et al., 2014). The authors suggested based on these findings, that difficulties of inhibition and hyperactivity in ADHD-C type may be characterized by these frontal–subcortical abnormalities. The authors found differences in both ADHD types relative to controls with abnormalities in temporo–occipital regions in ADHD-I and in the frontal–subcortical, fronto-limbic and temporo–occipital regions in ADHD-C. Connectomic disturbances were reported in a study by Hong et al. (2014), which applied network-based statistic (NBS) analysis of FA values to measure connectivity differences in specific brain sub-networks in 39 ADHD-C and 26 ADHD-I participants and 26 controls, aged 6–15 years. Decreased FA in the right lateralized network involving 17 brain regions was shown in ADHD-C relative to ADHD-I, involving the superior frontal gyrus, anterior cingulate gyrus, and supplementary motor areas, which are associated with attention orienting to external stimuli and executive functioning. This was further validated by observation of omission error and reaction time scores for continuous performance tasks to be negatively correlated with FA in regions involving the superior frontal gyrus, anterior cingulate gyrus, and supplementary motor area in ADHD-C than ADHD-I.

### Functional MRI and ADHD Subtypes

Overall, seven studies were available to review which have used fMRI to examine functional activation and connectivity differences between the ADHD-C and ADHD-I subtypes. Of these seven studies, two utilized task-based fMRI (Solanto et al., 2009; Orinstein and Stevens, 2014) and five were rs-fMRI studies (Fair et al., 2012; dos Santos Siqueira et al., 2014; Ahn et al., 2015; Pikusa and Jonczyk, 2015; Sanefuji et al., 2016). Various task-based paradigms were utilized in the two studies reviewed, which involved a three-stimulus auditory oddball attention task (Orinstein and Stevens, 2014), and the Go/No-Go response inhibition task (Solanto et al., 2009). Findings of differing activation patterns from these task-based fMRI studies distinguishing the two subtypes showed both increased and reduced activation in frontoparietal regions in ADHD-I and in the occipital–parietal regions in ADHD-C depending on the nature of the functional task. Solanto et al. (2009) used the Go/No-Go task to assess differences in inhibitory control in children, aged 7–13 years, with ADHD-I and ADHD-C types. The study reported increased activation in regions involving the frontoparietal and ventral attention networks in ADHD-I relative to ADHD-C. However, in ADHD-C, increased activation in the cuneus was found, known to be associated with higher-level visual and spatial attention processing (Solanto et al., 2009), which is linked to the visual network. Orinstein and Stevens (2014) compared hemodynamic responses to brain activation during target and novel stimuli, auditory oddball attention tasks in adolescents, aged 12–18 years, and found lower activation in ADHD-I involving regions linked to the cingulo–frontoparietal (CFP) attention network, relative to ADHD-C.

Overall, the five rs-fMRI studies available to review, all utilized data from the “ADHD-200 database” (Bellec et al., 2017), demonstrated significant differences between the ADHD-C and ADHD-I subtypes (Fair et al., 2012; dos Santos Siqueira et al., 2014; Ahn et al., 2015; Pikusa and Jonczyk, 2015; Sanefuji et al., 2016). Consistent with the findings of task-based fMRI studies (see above), patterns of aberrant connectivity within the CFP attention and DMN networks between the two subtypes are reported in rs-fMRI studies, which further support the role of the CFP attention and DMN as key networks in ADHD pathophysiology. Sanefuji et al. (2016) applied statistical clustering to define data-driven subtypes using Conners' parent-rated clinical scores to classify participants ADHD-C (*n* = 68), 53 ADHD-I (*n* = 53), and 44 ADHD-HI (*n* = 44) compared to 170 controls. They found increased resting state connectivity in the right ventral attention network in ADHD-I relative to the ADHD-C and ADHD-HI types and also controls (Sanefuji et al., 2016). rs-fMRI studies incorporating graph theoretical analysis have observed atypical connectivity patterns in the sensorimotor and DMN in ADHD-C and the frontoparietal network and cerebellar regions in children with ADHD-I (Fair et al., 2012; dos Santos Siqueira et al., 2014). In one of the first studies to utilize graph network analysis on functional connectivity data, Fair et al. (2012) investigated possible neural differences between the ADHD-C and ADHD-I subtypes. Based on a sample of 80 ADHD-I, and 112 ADHD-C participants, and 455 neuro-typical controls, aged 7–14 years, the authors also found alterations of the DMN and the insular cortex in ADHD-C and aberrant network properties for the dorsolateral prefrontal cortex and cerebellum in ADHD-I. Deficits in language and communication processing are often associated in ADHD and were examined in a study by Pikusa and Jonczyk (2015) using fractional amplitude low-frequency fluctuations (fALFF) using rs-fMRI, between 158 ADHD-I, 109 ADHD-C, and 478 controls. While no significant differences between subtypes were found, ADHD-I had lower fALFF in Brodmann area 44, while ADHD- C had lower fALFF in area 45, both relative to controls (Pikusa and Jonczyk, 2015). Interestingly, even though both areas are part of the Broca region, it appears their functional roles differ. Utilizing a sparse reduced rank (SRR) spatial–temporal modeling framework in the frequency domain on rs-fMRI data from 76 ADHD-C, 44 ADHD-I, and 99 controls, aged 7–18 years, Ahn et al. (2015) revealed different activations between the two subtypes with higher power spectra in the right middle and inferior frontal gyrus in ADHD-C and higher power spectra in the left middle frontal gyrus and cingulate gyrus, right insula, and right postcentral gyrus in ADHD-I.

## Discussion

This review integrates the results from 19 studies involving magnetic resonance imaging (structural, diffusion, and functional MRI) data to explore the neural mechanisms that underlie the combined and inattentive subtypes of ADHD. The goal was to synthesize these results and examine if these findings were consistent across different modalities and produced similar patterns. Furthermore, our second aim was to present these findings from a perspective of brain circuitry dysfunction and its role in understanding the subtypes of ADHD wherever possible. Overall, of these studies reviewed, findings support that a shift in understanding ADHD symptom deficits from solely region-based structural or functional abnormalities to a more holistic connectivity perspective focusing on brain network framework is necessary. While it may be expected that an overlap in abnormalities would occur given the shared core inattentive symptoms across the two most common subtypes, the combined and inattentive type, imaging results support distinct differences.

### Neuroimaging Features Underlying the ADHD Combined and Inattentive Types

#### Structural MRI Findings

In summary, the structural MRI studies reviewed produced equivocal results, with only one of these studies reporting volumetric differences between the combined and inattentive subtypes. Findings from these studies generally found no significant volumetric differences between the ADHD-C and ADHD-I subtypes for global and specific subregions of the basal ganglia. Furthermore, another research, which has examined volumetric differences in just one subtype comparably to controls also reveals mixed results. For example, relative to controls, de Mello et al. (2013) found reduced gray matter volume of the left medial frontal gyri, ACC, caudate, thalamus, and right postcentral gyrus in ADHD-I, yet another study by Carmona et al. (2009) observed no significant differences. Studies investigating ADHD-C have shown greater hippocampal and left orbitofrontal cortex volume (Plessen et al., 2006); however, reduced ventral–striatal volumes (Carmona et al., 2009) and smaller global gray matter volumes in the frontal, parietal, temporal, and occipital lobes (Batty et al., 2010) have also been reported relative to controls. Certainly, more studies are required to ascertain the presence or lack of volumetric differences to address the methodological differences of these studies. As a general limitation, the small sample sizes (with the exception of one study using the ADHD-200 database) of the studies included in this review (range of 15 to 33 per subtype group) may have underpowered the analyses, imposing limitations to delineate confounding effects of medication and comorbidity (Horga et al., 2014). Although the inclusion of research evidence relating to the effect of medication on structural neuroanatomical substrates are beyond the scope of this review, studies have generally observed smaller ACC and caudate gray matter volumes and deviant cerebellar morphology in treatment naïve relative to treatment experienced in samples of ADHD-C participants and controls (Bussing et al., 2002; Semrud-Clikeman et al., 2006, 2014b; Bledsoe et al., 2009; Villemonteix et al., 2015). Similarly, structural asymmetry, i.e., differences in size between left/right brain regions, which has been shown to be a feature in ADHD, was also found to be different in unmedicated vs. medicated patients (Douglas et al., 2018). It is likely that the medication disparity between the subtypes could be driving some of the neural findings.

While volumetric data preclude any interpretations related to brain networks, only one study, that of Saad et al. (2017), has so far highlighted findings of differential structural network properties between the ADHD-C and ADHD-I subtypes using graph theoretical analyses. Saad et al. (2017) demonstrated distinguished organizational profiles between subtypes using covariance network measures using volume, which is considered as characteristic of functional connectivity and in support of networks identified in ADHD subtypes from functional imaging studies (Fair et al., 2012), i.e., the limbic, visual, and ventral attention networks associated with ADHD-I, and the motor, frontoparietal, and DMN networks in ADHD-C, highlighting the role of network organization as an important factor in understanding ADHD subtype pathophysiology.

#### Diffusion Tensor Imaging Findings

Overall, of the four studies reviewed, aberrant white matter brain microstructure between the subtypes was reported, though with varied differing results mostly driven by differences in methodological or participant characteristics. For example, despite both studies employing a TBSS approach, opposing findings of increased RD may be due to the treatment-naïve status and exclusion of comorbidity, except ODD, from the Ercan et al. (2016a) participant sample from the Svatkova et al. (2016) findings. However, despite the paucity of DTI studies examining subtype differences, emerging evidence from DTI studies in ADHD report similar results. Specifically, fiber tracts differed in regions involving the cerebellum, frontostriatal and frontoparietal regions in ADHD-C compared to ADHD-I (Hong et al., 2014; Lei et al., 2014; Ercan et al., 2016a), and also for each subtype in comparison to controls. Also, results from studies investigating basal ganglia and thalamic connectivity have found lower FA values in ADHD-C relative to controls (Ashtari et al., 2005; Xia et al., 2012). Fall et al. (2015) indirectly support these findings reporting mean reaction times correlated with MD values in the striatum and thalamus in ADHD-C compared to controls during a flanker task. These studies collectively seem to support white matter alterations related to the motor network for the ADHD-C subtype. These are consistent with cerebellar abnormalities also observed in regional networks measured by structural gray matter data reviewed above. Furthermore, support for these distinct structural and white matter connectivity disturbances between the ADHD-C and ADHD-I subtypes relative to frontal, striatal, and cerebellar regions are consistent with functional deficits also observed in these corresponding subtypes in fMRI data (see below).

#### Functional MRI Findings

Notably, there were more resting-state functional connectivity studies than task-based studies available for review. Overall, the key findings from the fMRI studies reviewed indicate frontoparietal, cerebellar deficits, and aberrant CFP attention network patterns in ADHD-I, and frontostriatal thalamic and visual deficits in addition to aberrant DMN connectivity in ADHD-C. Interestingly to note, these rs-fMRI results produce an opposing pattern to the atypical cerebellar findings observed in the ADHD-C type in DTI and structural volume studies reviewed earlier. However, this is not a surprising finding in the inattentive type, as the role of the cerebellar system in modulating motor movement is suggested to extend its involvement of executive control and coordination of executive functioning (Faraone et al., 2015), which underpin the inattentive symptoms. Task-based findings of aberrant activations in brain regions underlie functional deficits concordant to clinical symptoms characteristic of the subtype and thus support pathophysiological differences between the ADHD-C and ADHD-I subtypes (Solanto et al., 2009; Orinstein and Stevens, 2014). Consistent with the findings of task-based fMRI studies, patterns of atypical DMN and CFP attentional networks are also supported by resting-state functional connectivity data (Fair et al., 2012; dos Santos Siqueira et al., 2014; Ahn et al., 2015; Pikusa and Jonczyk, 2015; Sanefuji et al., 2016), only strengthening the role of the CFP attentional and DMN as key networks in ADHD pathophysiology. Task-based studies investigating either the ADHD-C or the ADHD-I subtype, relative to controls, also provide supporting evidence for these aberrant functional differences in ADHD subtypes. Greater activation in the temporo–occipital and posterior brain regions were found in the hypothesized restrictive inattentive ADHD (ADHD-RI) type (Ercan et al., 2016b) and decreased activation in ADHD-I, relative to ADHD-C, in regions involving the cingulo–frontal–parietal attention (Orinstein and Stevens, 2014) has been reported. Studies examining the ADHD-C type showed task-dependent activation differences in regions involving the parietal, temporal lobes and basal ganglia, in line with the findings suggestive of fronto–striatal–thalamic deficits (Silk et al., 2005; Vaidya et al., 2005; Smith et al., 2006, 2008; Tamm et al., 2006; Rubia et al., 2007; Stevens et al., 2007; Vance et al., 2007; Fassbender et al., 2009; Iannaccone et al., 2015). Further, task-based fMRI studies comparing ADHD-C and controls have shown reduced activation and aberrant connectivity in regions associated with visual attention processing (Vance et al., 2007; Li et al., 2012). Comparably, using rs-fMRI data to examine the ADHD-C type relative to controls has also shown atypical cortico–striatal–thalamic circuitry (Mills et al., 2012; Dias et al., 2013) and the DMN (Wang and Li, 2015). Atypical connectivity of the DMN has also been observed in ADHD-I (Qiu et al., 2011), although, in comparison, the sample size of this study was considerably smaller. These aberrant connectivity patterns in these networks are concordant with symptoms associated with each ADHD subtype. That is, functional deficits in frontostriatal–thalamic circuitry and the DMNs are consistent with deficits of response inhibition, distractibility, impulsivity, goal-directed activity, and attentional performance in the ADHD-C type, whereas there is dysfunction involving the frontoparietal region, in addition, the CFP is consistent with deficits characteristics in the ADHD-I subtype including sustained attention and intrinsic motivation.

### Brain Circuitry Dysfunction Underlying the ADHD Combined and Inattentive Types

In summary, our review found consistent patterns across the imaging modalities in each subtype, which highlighted network organization differences with patterns of the DMN associated to ADHD-C and cingulo–frontoparietal (CFP) attention in ADHD-I. Additionally, while cerebellar abnormalities were found in the ADHD-C type across measures of brain structure, rs-fMRI deficits involving the cerebellar network were also reported for the ADHD-I type. There were also findings, which were consistent across some, but not all, imaging modalities involving the cerebellum and motor networks in ADHD-C, and differences involving the visual network in ADHD-I. Collectively, these findings might suggest that there are core differences in large-scale brain networks between the two ADHD subtypes. Variations in the presentation of the clinical symptoms of ADHD between the subtypes have been highlighted in a recent review by Bush (2010), acknowledging that despite shared clinical symptoms, differences in network organization may characterize the subtypes and better account for the varied presentation of clinical symptoms between the subtypes. Although deficits of inhibition and attention performance are observed in both the ADHD-I and ADHD-C subtypes, neurally, these may arise due to different mechanisms possibly driven by direct deficit in those neural circuits revealed from the findings. That is to say, the clinical deficits in ADHD-I may be driven by disruptions in the CFP attention network that plays a role in attention, cognition, executive function, motor control, response inhibition, working memory, and reward/motivation (Bush, 2010), whereas an indirect greater suppression in these processes due to dysregulation and/or interference from the DMN, which acts as a state regulation mechanism by suppressing activity during cognitive demand and increasing activity during resting states (Raichle, 2015), may drive these same clinical deficits in the ADHD-C type, i.e., issues with regulation of inhibition and attentional performance during goal-directed activity, motivational effort, and sustained attention (Fair et al., 2012; Mohan et al., 2016; Saad et al., 2017).

Growing evidence from both functional and structural connectivity studies highlight brain connectivity differences between ADHD subtypes, which extends support for key brain networks that may underlie the combined and inattentive types (Qureshi et al., 2016). Disorganization of these key neural networks in ADHD gives rise to the symptoms that clinically categorize the inattentive and hyperactive/impulsive symptoms in ADHD. To advance the neurobiological framework of ADHD requires a greater understanding of the underlying brain-based pathogenesis complementary to a neuropsychological categorical system. Based on connectivity and network findings from MRI studies and considering equivocal findings, future studies should also integrate both structural and functional connectivity data. Employing a connectome approach is important for the pursuit of brain network-based markers, for an improved understanding of pathophysiology and treatment outcomes in ADHD (Cao et al., 2014). Dysfunction or dysregulation may not be driven by a single neural mechanism, but instead reflects a multi-layered framework that is better represented by a network approach (Williams, 2016). An improved understanding of the regulation of key networks in ADHD has already been suggested to substantiate the ADHD neurobiological framework (Fair et al., 2010); such an approach would further help us understand the clinical presentations of this disorder.

Therefore, a brain network-based approach not only can help to advance the neurobiological ADHD framework but could also be useful to further evolve dimensional constructs of ADHD neurobiology. The RDoC framework, which incorporates a dimensional analysis approach, could provide an alternative diagnostic construct based on neurobiological measures and observable behavior to address the primary symptoms that are defined by DSM diagnostic categories (Kelly et al., 2017). However, the DSM categorical diagnostic approach currently remains the benchmark for diagnosis in clinical settings, and as such, studies examining the neurobiological features of DSM-based ADHD subtypes offer an opportunity to inform improved clinical approaches. For example, the underlying mechanisms of ADHD are not simply driven by dysfunction in cognitive processes but also involve the influence of environmental factors on the variation and severity of behavioral symptoms (Rommelse and de Zeeuw, 2014), such as early parental attachment issues, which can be accounted for as part of the DSM-based diagnostic approach. Irrespective of diagnostic approach, whether DSM or RDoC, examining neurobiological mechanisms of ADHD subtypes hold benefit to further inform the development of causal relations of these altered neural pathways and its implications to symptoms and treatment. The focus is not on a dispute of diagnostic criteria, but to direct investigations that may provide greater clarity of the underlying subtype pathophysiology.

Furthermore, understanding the key brain networks implicated in the combined and inattentive ADHD subtypes may lead to the availability of personalized treatment interventions based on “actionable connectivity metrics” providing an opportunity for improved clinical outcomes (Faraone et al., 2015; Williams, 2017). For example, studies have shown methylphenidate to suppress activity in the DMN, which may be associated to improved performance on continuous performance tasks (Silberstein et al., 2016). Therefore, consistent findings of disrupted DMN network organization may translate to targeted pharmacological treatment for the ADHD-C type. Furthermore, findings have suggested the role of meditation in regulating the DMN, and this may also form part of the treatment recommendation for the combined type, whereas, the role of the CFP in support of attention and executive functioning, which has been shown to be atypical in the ADHD-I type, has been shown in depression study to respond to non-pharmacological treatment involving cognitive behavioral therapy (Williams, 2017).

Finally, another important aspect to consider between the two subtypes is the trajectory of symptoms with neurodevelopment. The onset of attentional issues in the inattentive type compared to the combined type has been found to develop somewhat later, in addition to diminishing hyperactive/impulsive symptoms with increased age, which also supports the suggestion of underlying neurobiological differences between the subtypes with age (Willcutt et al., 2012). We did not find any existing study that evaluates how neurodevelopment could play a role in neural mechanisms underlying the two subtypes, and this should be evaluated in future work.

### Limitations

There are several limitations of this review to consider against the interpretation of these findings. We excluded the three unpublished articles (“gray literature”) from this systematic review, as conference abstracts typically are not rigorously peer reviewed, and the results reported tend to be preliminary and not finalized in preparation for conference presentation, with difficulty to effectively assess the details of the study in depth (Scherer and Saldanha, 2019). Notably, comparability of these results from the studies included in this review is limited due to the heterogeneity in the neuroimaging methodology and diagnostic assessment measures used. Moreover, these studies often involve small sample sizes and represent huge variations (e.g., treatments, age ranges) in cohorts, which are a common limitation across the available literature in this area of ADHD research. Overall, a small number of studies are available in this field, which remains a general limitation in ADHD research. Furthermore, the literature in this field could be particularly biased as studies using different methodology on the same dataset have produced different results. Hence, it is likely that the lack of findings in the studies could be related to the lack of more sophisticated methods employed. An example of methodological differences between two studies producing differing results is observed in the Anderson et al. (2014) study, which used sophisticated methods, and found the DMN to be associated with the ADHD-I type relative to ADHD-C, ADHD-HI type, and controls, while the DMN was found to be associated with the ADHD-I type relative to ADHD-C, ADHD-HI type, and controls, and was found to distinguish the ADHD-C group from ADHD- I, using the same ADHD-200 dataset in the Fair et al. (2012) study. Similarly, other studies using the same dataset were unable to identify neural differences between subtypes (Colby et al., 2012; Douglas et al., 2018). Thus, consideration of the sensitivity of measures to identify biological differences between the ADHD types is warranted as this may bias the results so far in identifying the subtypes. For this review, the inclusion of studies was based on the goal of conducting a systematic review of findings from neuroimaging measures between the two subtypes to assess whether these results were equivocal or not across different neuroimaging modalities. Our focus is on whether using clinically categorically subtype diagnostic types could be distinguished based on brain organization, which is suited to this type of systematic review approach. While we acknowledge the value of a dimensional approach, that is, to explore the possible associations between clinical symptoms and MRI structural or functional properties; however, this is beyond the scope of this review. Also, studies specifically looking at the hyperactive/impulsive subtype only are underrepresented in the literature, and therefore, a comparison of findings across all three subtypes is not possible. Because of the heterogeneity of the participant samples, diagnostic assessment measures used, and imaging modalities, a meta-analysis was not feasible and beyond the scope of this qualitative review. As the focus of this review included pediatric studies only, there were no adult ADHD studies reviewed. Medication history and comorbidity in these studies are difficult to control and may bias the findings (He et al., 2015). An important consideration relates to the age range utilized in these studies, which relate to significant neurodevelopmental periods and that by grouping these participants often into one large age band may confound the results due to changes expected at different maturational periods (Nakao et al., 2011). Another important consideration involves gender variation in subtype diagnosis, that is, where ADHD-C is typically diagnosed in males, and the underrepresentation of females in imaging studies that may explain differences in these findings (Rosch et al., 2018; Rubia, 2018). The participants from the included studies across both the ADHD-I and ADHD-C types are characterized by a majority of male cohort. Last, limitations exist in understanding the specificity of these findings relative to other disorders where the same networks are found implicated. That is, despite the crossover of findings of alterations in the DMN in other conditions such as depression, it is possible that these similar findings within the ADHD population are most likely driven by the co-occurring pathology of depression and anxiety in ADHD, of which such symptoms are linked to functional roles of regions involving the DMN.

## Conclusion

To the best of our knowledge, this is the first systematic review to integrate findings of magnetic resonance imaging (structural, diffusion, and fMRI) measures of the clinical subtypes of ADHD. Our primary objective was to examine these findings and present a summary of the literature to explore the neurobiological mechanisms that may underlie these subtypes and shed insight on possible neurobiological distinction underlying differing symptoms. To summarize, the results from the reviewed studies examining the subtypes of ADHD across neuroimaging measures lend further support toward the view of brain circuitry dysfunction to inform the ADHD neurobiological framework. Furthermore, the value of understanding brain network organization and connectivity may help us to better conceptualize the ADHD presentation types and their symptom variability.

## Author Contributions

JS was responsible for substantial contributions to the conception or design of the work or the acquisition, analysis or interpretation of data for the work, as first author. MK and KG both were involved in revising the review paper critically for important intellectual content and to ultimately provide approval for publication of the content. All authors contributed to the article and approved the submitted version.

## Conflict of Interest

KG has received honoraria from Shire. JS has received honoraria for educational seminars from Shire. The remaining author declares that the research was conducted in the absence of any commercial or financial relationships that could be construed as a potential conflict of interest.
